# The comparability of the universalism value over time and across countries in the European Social Survey: exact vs. approximate measurement invariance

**DOI:** 10.3389/fpsyg.2015.00733

**Published:** 2015-06-04

**Authors:** Florian Zercher, Peter Schmidt, Jan Cieciuch, Eldad Davidov

**Affiliations:** ^1^Department of Political Science, University of GiessenGiessen, Germany; ^2^University Research Priority Program “Social Networks”, University of ZürichZürich, Switzerland; ^3^Institute of Psychology, Cardinal Stefan Wyszyński University in WarsawWarsaw, Poland; ^4^Institute of Sociology, University of ZürichZürich, Switzerland

**Keywords:** European Social Survey, approximate vs. exact measurement invariance, Portrait Value Questionnaire, universalism, Bayesian estimation, cross-national research, repeated cross-sections

## Abstract

Over the last decades, large international datasets such as the European Social Survey (ESS), the European Value Study (EVS) and the World Value Survey (WVS) have been collected to compare value means over multiple time points and across many countries. Yet analyzing comparative survey data requires the fulfillment of specific assumptions, i.e., that these values are comparable over time and across countries. Given the large number of groups that can be compared in repeated cross-national datasets, establishing measurement invariance has been, however, considered unrealistic. Indeed, studies which did assess it often failed to establish higher levels of invariance such as scalar invariance. In this paper we first introduce the newly developed approximate approach based on Bayesian structural equation modeling (BSEM) to assess cross-group invariance over countries and time points and contrast the findings with the results from the traditional exact measurement invariance test. BSEM examines whether measurement parameters are approximately (rather than exactly) invariant. We apply BSEM to a subset of items measuring the universalism value from the Portrait Values Questionnaire (PVQ) in the ESS. The invariance of this value is tested simultaneously across 15 ESS countries over six ESS rounds with 173,071 respondents and 90 groups in total. Whereas, the use of the traditional approach only legitimates the comparison of latent means of 37 groups, the Bayesian procedure allows the latent mean comparison of 73 groups. Thus, our empirical application demonstrates for the first time the BSEM test procedure on a particularly large set of groups.

Over the last decades, considerable research on values has taken place (Hitlin and Piliavin, 2004). These theoretical and empirical research contributions have been inspired especially by Inglehart and his colleagues (Inglehart, 1977; Inglehart and Welzel, 2005) and Schwartz and colleagues (Schwartz, 2003; Schwartz et al., 2012). Inglehart's value measurement instruments have been implemented in the World Value Survey (WVS), whereas a short version of Schwartz's Portrait Values Questionnaire (PVQ) with 21 items has been included in the European Social Survey (ESS). Comparisons of the two theoretical conceptions and the measurement instruments based on them were undertaken by Datler et al. (2013) and Beckers et al. (2012).

To date, the PVQ has been the object of extensive comparative research in the social sciences. Studies have focused, for example, on the relation between values and political behavior, left-right orientation, attitudes toward immigration, attitudes toward homosexuality and sociodemographic characteristics (Davidov et al., 2008, 2014b; Piurko et al., 2011; Meuleman et al., 2012; Schwartz et al., 2012; Kuntz et al., 2015) by making use of increasingly available cross-national data sources, such as the ESS or the WVS. The cross-national orientation in the study of values offered the advantage of introducing a stricter test of propositions (Popper, 2005), thereby expanding our knowledge about the validity of theories in different societies and allowing us to acquire insights into macro-micro effects (Opp, 2011). However, in comparative research, the issue of comparability across countries must be addressed (Davidov et al., 2014a). Respondents in different countries may understand survey questions in various ways (Latcheva, 2011; Braun et al., 2013) or respond in systematically different ways to the same questions (Harkness et al., 2010). This may lead to biased means, factor loadings and regression coefficients. Therefore, the assumption of cross-cultural measurement invariance needs to be tested (Meredith, 1993; Vandenberg and Lance, 2000; Davidov and Siegers, 2010; Millsap, 2011; Sarrasin et al., 2012; van de Schoot et al., 2012; Davidov et al., 2014a).

Davidov et al. (2008) and Davidov (2008, 2010) tested the measurement invariance properties of values across countries in three rounds of the ESS and could establish only metric invariance within the rounds across most countries and longitudinal scalar invariance within countries^1^. However, it remains to be answered if value measurements are invariant both across countries and over time and whether such an extensive test is feasible with real data. After all, various researchers who use values as explanatory or as explained constructs wish to test propositions referring simultaneously to different countries (“the cross-cultural aspect”) and time points (“the dynamic aspect”). Such an endeavor requires that measurement invariance is given simultaneously over time and across countries. However, such a measurement invariance test has not been performed in the past. Moreover, such a test becomes increasingly important considering the continuous growth in the number of countries *and* time points in the large data-generating programs mentioned before. Thus, our research question is whether it is feasible to test and establish measurement invariance across a very large number of groups.

In the current study we would like to focus on the universalism value because it is the only value which was measured in the PVQ-21 with three (rather than only two) items, thus allowing us to control for all forms of random and nonrandom measurement errors (Bollen, 1989). Furthermore, this universalism scale has also been used in a considerable number of empirical studies using ESS data (Jowell et al., 2007; Beierlein et al., 2012; Davidov et al., 2012; Saris et al., 2013) and other datasets (Schwartz et al., 2012; van de Schoot et al., 2012). We will examine its simultaneous comparability across 15 countries and six time points using the new procedure for assessing approximate invariance using Bayesian estimation (van de Schoot et al., 2013). To the best of our knowledge, no previous study has assessed invariance across so many groups simultaneously^2^. We will demonstrate the application of the two approaches on the same large set of time/country groups. Given previous findings, we expect to find metric invariance at best for the universalism scale but no scalar invariance across countries using the traditional exact method. However, we expect to establish scalar invariance at least for a subset of countries using the approximate approach.

We begin by briefly presenting the traditional exact approach and then describe the new approximate approach to test for measurement invariance across groups. Next, we describe our data and the three items that measure universalism. In the empirical part we report the results of the two approaches to test for invariance. We finalize with a discussion of the pros and cons of the traditional exact approach vs. the approximate approach to test for measurement invariance in cross-national research.

## The traditional approach to measurement invariance testing: multi-group confirmatory factor analysis (MGCFA)

Multi-group confirmatory factor analysis (Jöreskog, 1971; Bollen, 1989; Brown, 2006) has been the most common method used to test for measurement invariance. There are three distinct and hierarchically ordered levels of measurement invariance. Each level is defined by the parameters constrained to be equal across groups. The first and lowest level is configural invariance (Horn and McArdle, 1992; Meredith, 1993; Vandenberg and Lance, 2000). Configural invariance requires that each construct is measured by the same items. The second level is metric invariance, and it guarantees that the measured construct essentially has the same meaning in the different groups under study. Full metric invariance is tested by constraining the factor loadings to be equal across the groups to be compared (Vandenberg and Lance, 2000). If full metric invariance is established, a one-unit increase in the latent construct has the same meaning across groups. Subsequently, covariances and unstandardized regression coefficients may be meaningfully compared across samples (Steenkamp and Baumgartner, 1998). However, it is still uncertain whether the construct is measured on the same scale (Horn and McArdle, 1992; Steenkamp and Baumgartner, 1998; Vandenberg and Lance, 2000). Scalar invariance requires, in addition, that the intercepts are equal across groups. It is tested by constraining both the factor loadings and the intercepts to be equal across the groups to be compared (Vandenberg and Lance, 2000). If full scalar invariance is established, also the means may be meaningfully compared across groups (Steenkamp and Baumgartner, 1998).

Below, the corresponding three sets of constraints for the three levels of invariance are defined for a particular item in a one-factor case for individual *i* in group *j* (see Muthén and Asparouhov, 2013).

(1) $$\begin{array}{l} \text{Configural invariance }y_{ij}=v_{j}+\;\lambda_{j}f_{ij}+\varepsilon_{ij}\\ \;E\left(f_{i}\right)=\;\alpha_{j},V\left(f_{j}\right)=\;\psi_{j} \end{array}$$

Where v is a measurement intercept, λ is a factor loading, f is a factor with mean α and variance Ψ, and ε is a residual with mean zero and variance θ, uncorrelated with *f*. The configural model has subscript *j* for both intercepts and loadings.

(2) $$\begin{array}{l} \text{Metric invariance }y_{ij}=v_{j}+\lambda f_{ij}+\varepsilon_{ij}\\ \;E\left(f_{i}\right)=\;\alpha_{j}=\;0,\;V\left(f_{j}\right)=\;\psi_{j} \end{array}$$

The metric model drops the subscript *j* for the loadings because they are assumed to be equal.

(3) $$\begin{array}{l} \text{Scalar invariance }y_{ij}=v\;+\;\lambda f_{ij}+\varepsilon_{ij}\\ \;E\left(f_{i}\right)=\;\alpha_{j},V\left(f_{j}\right)=\;\psi_{j} \end{array}$$

The scalar model drops the subscript *j* for both intercepts and loadings because they are assumed to be equal^3^.

In practice, it is particularly difficult to reach full scalar invariance. Variations in the way respondents react to questions or systematic response biases such as social desirability or acquiescence (Billiet et al., 2003; Oberski et al., 2012), which may be individually or culturally determined, could possibly distort responses to the extent that scalar invariance will not exist in most empirical applications (Davidov et al., 2014a). There have been basically two major approaches to handling the issue of measurement noninvariance (Jouha and Moustaki, 2013; van de Schoot et al., 2013; Davidov et al., 2014a):

1. Ignoring it. This is what the overwhelming majority of researchers have done as is evident in publications using cross-national and multigroup data, repeated cross-sections and panel data (see Davidov et al., 2014a). This line of literature has typically used sum scores instead of first testing whether the assumption of invariance can be supported by the data. As Steinmetz (2013) demonstrated in a Monte Carlo study, the use of sum scores is not an adequate procedure without invariance testing, as sum score differences are only warranted in conditions of full measurement invariance.
2. Byrne et al. (1989) and Steenkamp and Baumgartner (1998) proposed the concept of *partial invariance* as a sufficient condition for meaningful cross-group comparisons. This approach has become a standard approach among various researchers. Partial invariance is given if the parameters of *at least* two indicators per construct (i.e., loadings for partial metric invariance and loadings plus intercepts for partial scalar invariance) are equal across groups. Several scholars rely on partial invariance when comparing countries, cultures or other units of analysis. However, even partial scalar invariance may often be rejected.

Three common procedures in the MGCFA literature which rely on global fit measures have been proposed to evaluate whether measurement invariance is established:

1. To rely on the chi-square difference test and compare the configural, metric and scalar invariance models, which form nested models (Jöreskog, 1978; Bollen, 1989; Meredith, 1993; Brown, 2006). According to this procedure, the chi-square difference test is used to assess the correctness of the model. However, the use of the chi-square difference test has been criticized because of its sensitivity to sample size (among other reasons) (Jöreskog, 1993; Cheung and Rensvold, 2002).
2. To use cut-off values for the *difference* in the comparative fit index (CFI), the root mean square error of approximation (RMSEA) and the standardized root mean square residual (SRMR) (Chen, 2007; for alternative cut-off values see Meade et al., 2008). According to this procedure, if the change in model fit is smaller than the criteria proposed in the literature, measurement invariance for that level is established. According to the results of (Chen's, 2007) simulation study, the following recommendations have been proposed:
  - If the sample size is larger than 300, metric noninvariance is indicated by a change in CFI larger than 0.01 supplemented by a change in the RMSEA larger than 0.015 or a change in SRMR larger than 0.03 compared with the configural invariance model.
  - Scalar noninvariance is evidenced by a change in CFI larger than 0.01 supplemented by a change in RMSEA larger than 0.015 or a change in SRMR larger than 0.01 compared with the metric invariance model.
3. The third procedure suggests employing the Akaike information criterion (AIC) and the Bayesian information criterion (BIC) information theoretic measures to compare the configural, metric and scalar invariance models (Kass and Raftery, 1995). Following the criteria proposed by Kass and Raftery (1995), a very strong difference is indicated when the AIC or BIC difference is greater than 1^4^.

Since empirical tests often fail to establish measurement invariance based on these criteria, it has been argued that the criteria for testing measurement invariance may be too strict (Muthén and Asparouhov, 2013) and that more liberal criteria should be used to assess approximate (rather than exact) measurement invariance.

## The bayesian approach to test for approximate measurement invariance

Recently, Muthén and Asparouhov (2013) and van de Schoot et al. (2013) proposed an alternative approach to test for measurement invariance by applying approximate Bayesian measurement invariance testing. The exact procedure, which constrains factor loadings and intercepts to be exactly equal to establish measurement invariance, is very restrictive and rarely establishes invariance (Jouha and Moustaki, 2013; van de Schoot et al., 2013). Approximate measurement invariance permits “small” differences between parameters (van de Schoot et al., 2013). The parameters specified in a Bayesian approach are considered to be variables, and their distribution is described by priors. The assignment of prior distributions to unknown parameters reflects the researcher's uncertainty about them regardless of whether one conceives of a parameter as having one true value or not (Levy and Choi, 2013). Such uncertainty may be applied for various parameters both in single-group CFA and MGCFA. In invariance testing one may assume that differences between parameters (factor loadings, intercepts) are approximately equal. Thus, we would allow the introduction of some uncertainty by specifying a small variance of, for example, 0.01 or 0.05 around the difference in factor loadings or intercepts (van de Schoot et al., 2013).

**Figure 1:**
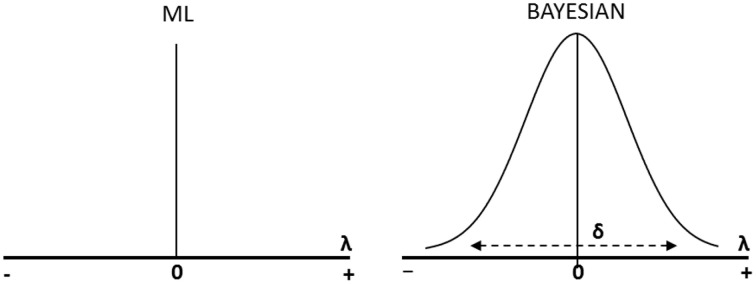
**Difference in parameter estimation between Maximum Likelihood (ML) and the Bayesian approach (see Muthén and Asparouhov, 2013; van de Schoot et al., 2013)**.

Figure 1 delineates the difference between the traditional exact approach to test for measurement invariance and the Bayesian approximate approach. In the traditional exact approach, the differences of factor loadings (λ) or intercepts (*v*) between groups are assumed to be exactly zero, while in the Bayesian approach the differences are assumed to be approximately zero with a mean of zero and some small variance delta (δ). Thus, we allow small variations in a given interval between the parameters as part of the measurement model^5^ (see also Kruschke et al., 2012; Muthén and Asparouhov, 2012, 2013; Levy and Choi, 2013). Simulations suggest that “small” variations may be allowed without risking invalid conclusions in comparative research (van de Schoot et al., 2013).

The difference between the traditional exact approach and the Bayesian approximate approach is also evident in the definitions of the confidence interval (used in the traditional exact approach) and the credibility interval (CI) (used in the Bayesian approximate approach). The confidence interval over an infinite number of samples taken from the population expresses that 95% of these contain the true population value. By way of contrast, the CI expresses that there is a 95% probability that the population value is within the limits of the interval.

A number of fit measures have been proposed to specifically assess Bayesian models (Gelman, 2003, 2013; Levy, 2011). These fit measures can detect if the actual deviations are larger than those allowed by the researcher in the prior distribution. First, the model fit can be evaluated based on the posterior predictive probability value (ppp). The ppp is computed by comparing two types of information: the discrepancy between the model and the observed data and the discrepancy between the model and the posterior predicted data (Levy and Choi, 2013, p. 597)^6^. According to Muthén and Asparouhov (2012) and van de Schoot et al. (2013), the ppp value of a model that fits the data should be nonsignificant, and if it is around 0.50, it indicates a well-fitting model.

A second fit measure refers to the CI for the difference between the observed and the replicated chi-square values. According to Muthén and Asparouhov (2012) and van de Schoot et al. (2013), the CI should contain zero. Finally, the BIC (Schwarz, 1978) and the deviance information criterion (DIC) (Spiegelhalter et al., 2002) were also proposed for the assessment of model comparison in a Bayesian framework (Kass and Raftery, 1995). BIC is computed using the following formula:

(4) $$BIC=\;-2\ell \left(\hat{\theta }|X\right)+p\;*\text{ ln}(n)$$

where $\ell \left(\hat{\theta }|X\right)$ is the maximized log-likelihood, *p* is the number of parameters, and *n* is the number of observations. Building on this tradition of comparing values of information criteria, Spiegelhalter et al. (2002) introduced the DIC:

(5) $$DIC=\bar{D\left(\theta \right)}+p_{D}=2\bar{D\left(\theta \right)}-D\bar{\left(\theta \right)}+2p_{D}$$

where *D*(θ) is the posterior mean of the deviation (negative of twice the log-likelihood function), *p_D_* is a complexity measure defined as the difference between the posterior mean of the deviance and the deviance evaluated at the posterior mean, *D*(θ)^7^.

Testing for approximate measurement invariance consists of two steps. The first identifies the noninvariant parameters while fitting the model to data. Noninvariant parameters are those parameters which are found to be outside of the “wiggle room” allowed for the parameter differences. In the second step the noninvariant parameters are freed and the model is recalculated (Muthén and Asparouhov, 2013; van de Schoot et al., 2013). In the next section we are going to provide a practical application by demonstrating a test for approximate invariance using ESS data.

## Method and data

For the analysis we employ data from the ESS measuring the universalism value (Schwartz, 2003; Schwartz et al., 2012)^8^. The ESS is a biannual cross-national European survey that is administered to representative samples from approximately 30 countries. Since its inception in 2002/2003, it has included questions that measure values in its core module. These questions have been repeated in each round and used extensively in cross-national research. In the present analysis we have included 15 countries which participated in all six rounds. Table 1 presents the sample sizes for each country/time point combination between 2002 and 2012.

**Table 1:** **ESS sample sizes for the selected 15 countries over six ESS rounds (2002–2012)**.

	**1st Round (2002/3)**	**2nd Round (2004/5)**	**3rd Round (2006/7)**	**4th Round (2008/9)**	**5th Round (2010/11)**	**6th Round (2012/13)**	***N***
Belgium	1899	1778	1798	1760	1704	1869	10,808
Switzerland	2040	2141	1804	1819	1506	1493	10,803
Germany	2919	2870	2916	2751	3031	2958	17,445
Denmark	1506	1487	1505	1610	1576	1650	9334
Spain	1729	1663	1876	2576	1885	1889	11,618
Finland	2000	2022	1896	2195	1878	2197	12,188
United Kingdom	2052	1897	2394	2352	2422	2286	13,403
Hungary	1685	1498	1518	1544	1561	2014	9820
Ireland	2046	2286	1800	1764	2576	2628	13,100
Netherlands	2364	1881	1889	1778	1829	1845	11,586
Norway	2036	1760	1750	1549	1548	1624	10,267
Poland	2110	1716	1721	1619	1751	1898	10,815
Portugal	1511	2052	2222	2367	2150	2151	12,453
Sweden	1999	1948	1927	1830	1497	1847	11,048
Slovenia	1519	1442	1476	1286	1403	1257	8383
*N*	29,415	28,441	28,492	28,800	28,317	29,606	173,071

Three items were used to measure the universalism value. Respondents were presented with a descriptive portrait of a person (gender matched), and they were requested to indicate to what extent they were similar to this person. The response scale ranged from 1 (*very much like me*) to 6 (*not like me at all*). These responses were reversed so that higher scores represented greater similarity to enable a more straightforward interpretation of the scores. The correlations between items were considerable and ranged approximately between 0.3 and 0.4. The rate of missing values for these items ranged from 4.0 to 4.2% only for each country/time point combination. Table 2 presents the item formulations.

**Table 2:** **Formulation of universalism items**.

“Now I will briefly describe some people. Please listen to each description and tell me how much each person is or is not like you. Use this card for your answer…”
Universalism Item1–“… She/he thinks it is important that every person in the world should be treated equally. She/he believes everyone should have equal opportunities in life.”
Universalism Item2–“… It is important to her/him to listen to people who are different from her/him. Even when she/he disagrees with them, she/he still wants to understand them.”
Universalism Item3–“… She/he strongly believes that people should care for nature. Looking after the environment is important to her/him.”

## Analytical strategy

### Testing for exact (full or partial) invariance

In the first step we performed six MGCFAs (one for each round) across 15 countries, and after that, the analysis was performed on all 15 countries and six rounds (with a total of 90 groups) simultaneously. In both cases, the full information maximum likelihood (FIML) procedure was used to deal efficiently with the problem of missing values (Schafer and Graham, 2002). We used the robustified maximum likelihood estimation procedure to deal with the ordered categorical character of the data^9^.

Each analysis contained assessments for configural, metric and scalar invariance, with the corresponding constraints for each level of the measurement invariance^10^. In a second step, when full measurement invariance was not established, we tried to assess partial measurement invariance. In order to establish partial scalar invariance (where at least two items are constrained to be exactly equal), the intercept of only one item was released, because partial scalar invariance requires that parameters of at least two items are constrained to be equal across all groups.

### Testing for approximate invariance

Following Muthén and Asparouhov (2013) and van de Schoot et al. (2013), we ran models with informative priors with a mean of zero and variances of 0.005, 0.01, 0.05, and 0.5 for the differences between factor loadings or intercepts across groups^11^. Next, we identified in each model with the different priors those factor loadings and intercepts which were different. In the next step we freed all parameters which were considerably different across groups and left the informative priors for all the other equality parameters intact (Muthén and Asparouhov, 2013). Table 3 summarizes the steps undertaken in each approach. These analyses were conducted on all ESS rounds and countries simultaneously.

**Table 3:** **Analytical steps for the exact and the approximate measurement invariance approaches**.

	**Traditional exact approach**	**Approximate approach**
Steps	1. Configural model 2. Metric model 3. Scalar model 4. Partial scalar model	1. Setting different informative priors for the cross-group differences of loadings and intercepts 2. Releasing (approximate) equality constraints (of loadings and intercepts) that are not supported by the data
Additional steps^12^	5. Deleting groups which are not fully or partially scalar invariant	3. Deleting groups which are not fully or partially approximately invariant

*As metric invariance could be established in the exact approach, we did not need to fall back to partial metric invariance*.

## Results

### The traditional exact approach

Table 4 presents the global fit measures of the accepted models after dropping countries using the traditional exact approach. The first part of the table presents the global fit measures of the accepted model in each round separately. The last part of the table presents the global fit measures for the accepted model in the simultaneous analysis across countries and rounds. After releasing the equality constraint on the intercept that had the highest modification index in most country/time point combinations (Byrne et al., 1989), we identified in the simultaneous analysis 53 country/time point combinations in which at least two items were noninvariant. These country/time point combinations had to be dropped from further analysis because, for these units, even partial invariance could not be established. For example, the items which measured the importance to understand different people and to take care of the environment were scalar noninvariant in Switzerland and Denmark at all measurement time points. Consequently, we dropped these countries from further analysis. Thus, in total, 37 of the country/time point combinations displayed partial exact scalar invariance^13^.

**Table 4:** **Global fit measures of the traditional exact approach**.

	**Chi^2^(df)**	**RMSEA**	**SRMR**	**CFI**	**Countries/Timepoints^14^**
**ROUND 1**					
Partial scalar	64.89 (24)	0.029	0.029	0.985	8
**ROUND 2**					
Partial scalar	53.28 (28)	0.022	0.027	0.992	9
**ROUND 3**					
Partial scalar	53.78 (27)	0.024	0.033	0.988	8
**ROUND 4**					
Partial scalar	87.43 (24)	0.040	0.041	0.978	8
**ROUND 5**					
Partial scalar	90.10 (21)	0.044	0.039	0.972	7
**ROUND 6**					
Partial scalar	69.26 (21)	0.034	0.036	0.980	7
**COUNTRIES AND ROUNDS SIMULTANEOUSLY**					
Partial scalar	348.23 (126)	0.031	0.035	0.983	37^15^

*RMSEA, root mean square error of approximation; SRMR, standardized root mean square residual; CFI, comparative fit index; the partial scalar model corresponds to step 5 in Table 3*.

Furthermore, we employed AIC and BIC comparisons of the metric invariance and partial scalar invariance models (see Table 5) in the separate analyses for each round and in the simultaneous analysis. Following the criteria proposed by Kass and Raftery (1995) to compare BIC differences, we can conclude that all differences between the metric and the partial scalar model, in a reduced number of countries, are very large.

**Table 5:** **AIC and BIC fit measures of the traditional exact approach^16^**.

		**AIC**	**BIC**
Round 1	Metric	232453.884	233335.682
	Partial scalar	133004.879	133373.601
Round 2	Metric	218452.710	219328.143
	Partial scalar	134813.330	135221.803
Round 3	Metric	222284.379	223163.765
	Partial scalar	106349.111	106687.021
Round 4	Metric	225469.593	226350.568
	Partial scalar	109976.943	110337.466
Round 5	Metric	226639.903	227520.419
	Partial scalar	98034.755	98344.903
Round 6	Metric	237036.130	237923.153
	Partial scalar	113273.097	113589.931
All rounds	Metric	1362329.608	1368665.132
	Partial scalar	537676.482	539559.803

The results have two important implications. On the one hand, findings of partial scalar invariance allow meaningful mean comparison across 37 country/time point combinations for the universalism construct. However, it is discouraging to find out that mean comparisons of the universalism value may be problematic in so many of the country/time point combinations. Next, we turn to the approximate invariance test.

### The bayesian approximate approach

Here, too, we first tested each round separately and then all rounds simultaneously^17^. Approximate measurement invariance across all countries was established in only two rounds (2002 and 2004). Next, as recommended by van de Schoot et al. (2013), we ran the model that included all time points and countries, using several prior variances to compare them. We released equality constraints on those loadings and intercepts which were different^18^. Finally, we deleted groups which were not approximately invariant. Table 6 reports the results for the model with a prior of 0.05 (Muthén and Asparouhov, 2013; van de Schoot et al., 2013).

**Table 6:** **Global fit measures for the approximate invariance test (mean = 0 and variance = 0.05)**.

	**ppp**	**ppp after releasing misspecified parameters**	**CI after releasing misspecified parameters**
90 groups	0.000	0.000	125.830–346.761
73 groups^19^	0.026	0.052	−10.834–171.115

*ppp, posterior predictive probability; CI, credibility interval*.

Accordingly, 73 countries/time points remained in the model. Thus, the results suggest that the exact and approximate measurement invariance approaches produce quite different findings. Whereas, partial approximate scalar measurement invariance was established in 73 ESS country/time point combinations, exact scalar measurement invariance was only established in 37 country/time point combinations. In other words, the approximate test allows us to perform mean comparisons of universalism across a very large set of countries and time points.

## Mean comparison

We compared the country means obtained from the MGCFA and Bayesian analyses with each other as well as with those based on the raw sum scores for the 73 comparable country/time point combinations. This was done by estimating mean scores based on the exact and approximate approaches and comparing them to each other and to those computed using the raw data. Finally, we estimated the correlation between the means computed in the country/time point combinations based on each of the three procedures.

**Figure 2:**
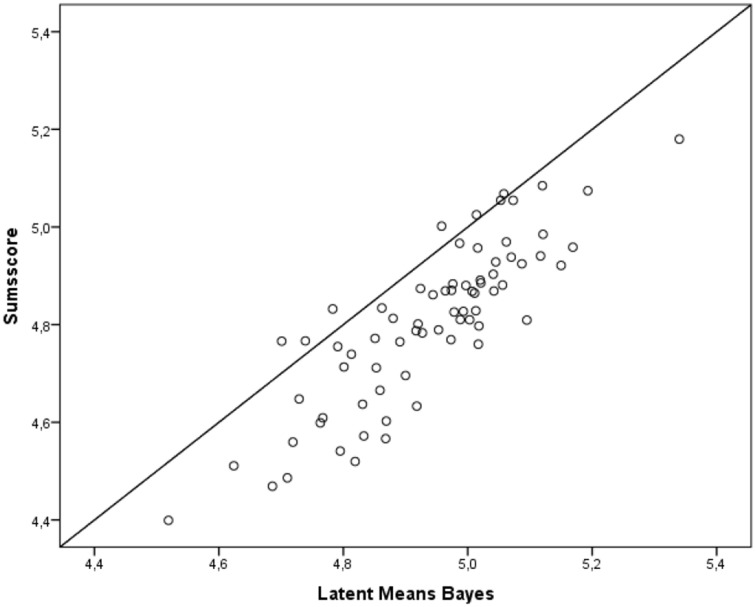
**Relationship between sum scores and scores based on the Bayesian estimation in 73 country/time point combinations**.

As Table 7 demonstrates, the correlation is highest between sum scores and the exact test (0.997), and the correlation between the Bayesian approximate test and the exact test (0.844) is lowest. Since the latent means from the approximate test are the only ones which rely on an acceptable model fit, we conclude that latent means based on the other approaches (the exact and the sum scores) are biased. Figure 2 presents the differences in the means between the sum scores and the scores from the approximate approach on a scatter plot. If the scores in the two methods were equal, they would all be on the diagonal. Stated another way, increased distance from the diagonal indicates increased differences between the scores.

**Table 7:** **Correlations between latent means computed using sum scores (1), the exact (2) and the approximate (3) measurement invariance models for 73 county/time points**.

	**Sum scores (1)**	**Exact test^20^ (2)**	**Approximate Bayesian test (3)**
1	1		
2	0.997^**^	1	
3	0.851^**^	0.844^**^	1

** *p < 0.01 (pairwise deletion)*.

**Figure 3:**
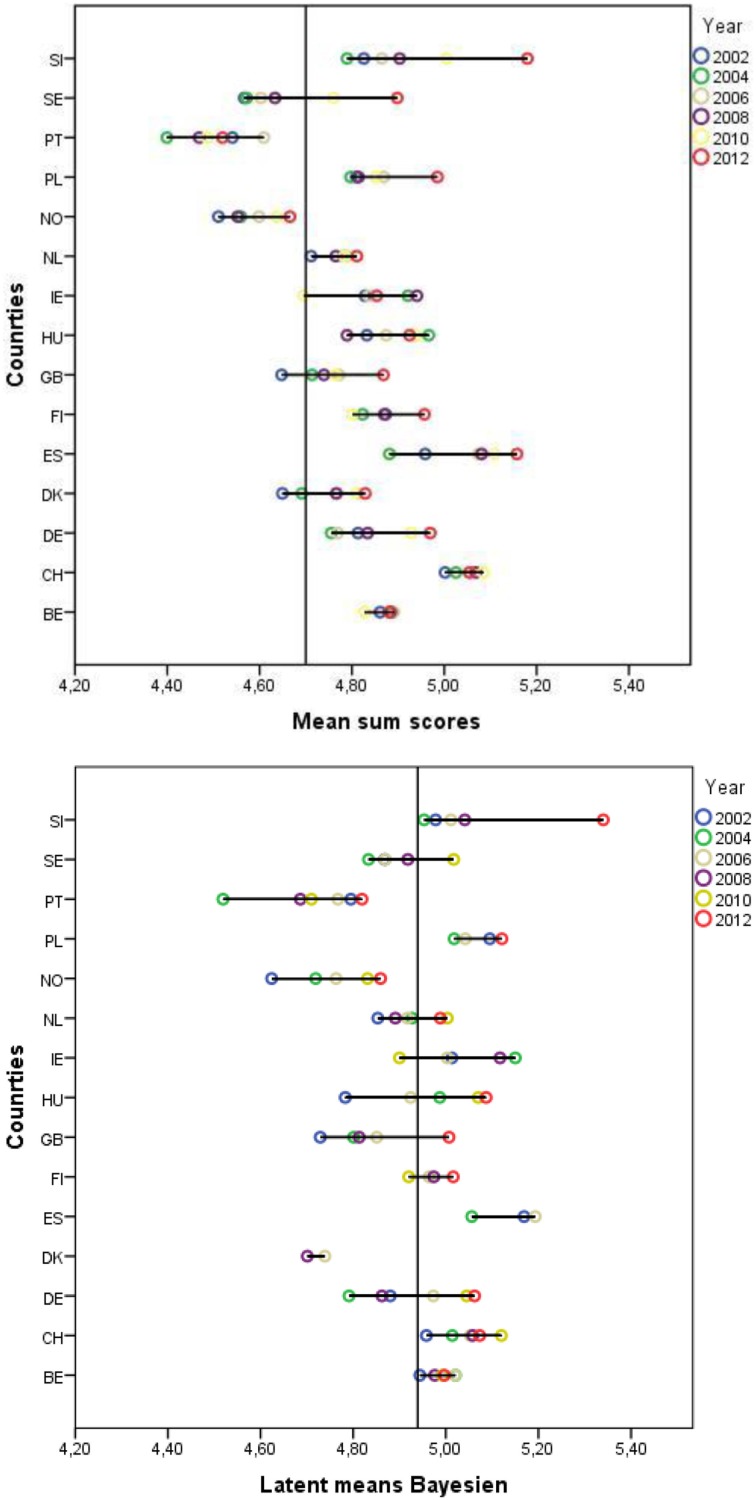
**Latent mean differences between 2002 and 2012 ^21^**.

Conclusions may also be biased when sum scores are compared for the same country longitudinally. Figure 3 presents the mean over time and within countries. For example, as Figure 3 demonstrates, when comparing the sum scores in Poland, one would assume that the means considerably increased between 2002 and 2012. However, based on the approximate approach, the data show that there was no mean difference between 2002 and 2012 for the universalism value scores in Poland. By way of contrast, the sum scores indicate no mean difference between 2002 and 2012 in Ireland. However, according to the approximate test, there was a slight increase in the universalism mean in Ireland between the two rounds. We thus conclude that if a researcher would draw conclusions based on the composite scores, either to compare countries with each other or to compare scores within the same country and over time, they might be misled by the scores and reach wrong conclusions. In Figure 3 one can see the variance of the latent means over the six time points. The length of the line shows the variation and the colored circles show the latent mean of universalism at in each round.

**Figure 4:**
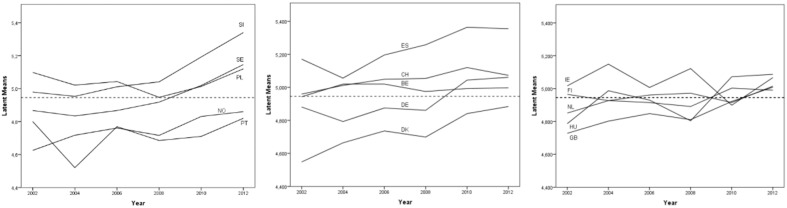
**Latent means over different time points and different countries ^22^**.

Finally, Figure 4 displays the mean development of universalism over time in each of 15 countries and how this compares to the overall mean level of universalism across all countries and rounds.

To visualize the latent means over different time points and different countries, we split the countries into three groups comprising five countries each. The straight, dotted horizontal line is the mean over all country/time point groups. The graphs depicted in Figure 4 suggest that the mean of universalism increases over time in most countries, while it remains more or less stable in Portugal, Ireland, Finland, and Belgium.

## Summary and conclusions

In most published cross-national studies, metric and scalar measurement invariance is implicitly assumed without testing this assumption. This may lead to biased mean comparisons and biased comparisons of covariances and regression coefficients (Vandenberg and Lance, 2000; Jouha and Moustaki, 2013; Oberski, 2014). However, the traditional estimation procedures used in MGCFA to test for measurement invariance and the corresponding global fit measures, especially in the case of scalar invariance assessments, mostly lead to a rejection of the assumption of even partial invariance. This often results in a considerable reduction in the number of countries and/or time points whose means can be meaningfully compared.

In the current study we assessed the comparability of the universalism value in six rounds of the ESS between 2002 and 2012 across all ESS countries, with 90 country/time point combinations in total. To the best of our knowledge, this is the first time in which so many groups are included in such a test. Using the traditional exact measurement invariance test procedure, metric invariance could be established across all country/time point combinations although partial scalar invariance could not, and we were required to drop almost two thirds of the countries/time points based on the reason that their mean scores on the scale might not be comparable.

To solve this problem we applied the newly proposed approximate measurement invariance procedure. In these analyses only 17 country/time point combinations had to be excluded. We could demonstrate that the assumption of (approximate) scalar invariance was tenable using this alternative procedure on the remaining countries. As a consequence, the latent means of universalism could be legitimately compared across many more countries and time points.

Having said that, we believe that the traditional exact approach should always be applied as a first step in invariance testing. After all, it could well be the case that measurements are exactly invariant and it is not necessary to apply approximate (rather than exact) constraints. Using only the exact approach may circumvent not only using the (technically more challenging) approximate approach but a practical problem we encountered while analyzing the data applying the approximate approach as well: Using it for so many groups with large sample sizes led to a computation time of between 12 and 16 h! However, where even partial *exact* measurement invariance does not hold, it would be useful to apply the approximate approach using Bayesian estimation (van de Schoot et al., 2013). This may be a relevant assessment especially in the case of comparisons of many groups such as in cross-national research with repeated cross-sections. As previous studies have demonstrated, in such cases it may be particularly difficult to establish full or partial (exact) scalar invariance.

It should be noted, however, that such a result in which so many country/time point combinations demonstrate approximate invariance may not necessarily be replicated with other data and other scales. Indeed, it could well be the case that both exact and approximate approaches fail to demonstrate cross-country and over time invariance. In other words, the approximate approach does not establish invariance where it is not given. It is, however, more liberal than the exact approach and may establish approximate invariance although the exact test fails to do so.

Future research may analyze various cross-national datasets with large samples to evaluate the approximate comparability of various scales and the practical usefulness of the approximate approach used here. In addition, it would be desirable if further simulation studies would be performed to evaluate which priors may be used in approximate invariance tests and which ppp values should be considered supportive for the assessed models. Such simulations could also explore how increasing the number of groups and the number of respondents in the groups may influence the results. This issue is particularly relevant because the number of groups (such as countries, cultural groups, language groups, etc.) in large data-generating programs such as the ESS, EVS, Eurobarometer, WVS, or the PISA study is continuously increasing. Furthermore, given that very often invariance cannot be established, it would be desirable if future studies would seek explanations for the absence of measurement invariance (see, e.g., Davidov et al., 2012, 2015). Finally, future research which includes a large number of groups may also apply other recent developments of testing for measurement invariance such as the alignment procedure (see, e.g., Muthén and Asparouhov, 2013) and examine the comparability of their findings to those of other more established approaches to test for invariance. Hopefully these methods and our empirical demonstration will encourage and support substantive researchers in their endeavor to conduct meaningful comparative research.

### Conflict of interest statement

The authors declare that the research was conducted in the absence of any commercial or financial relationships that could be construed as a potential conflict of interest.

## Footnotes

^1^For an invariance test of a new scale to measure human values, see Cieciuch et al. (2014a,b).

^2^In the study of van de Schoot et al. (2013), only a small number of groups was studied. Cieciuch et al's. (2014a) studies contained eight groups, and Davidov et al. (2015) contained 15 groups in six separate tests. None of these studies performed a simultaneous test over countries and time points, which would have led to a much higher number of groups.

^3^In the Analytical Strategy section we shortly describe our approach to identify the models.

^4^A more detailed analysis of the issue of robustness against violations of metric and scalar invariance is given in Jouha and Moustaki (2013), Oberski (2014), and Meuleman (2012). See also Saris et al. (2009) for an alternative procedure to assess whether exact measurement invariance is given which relies on identifying local misspecifications while taking the power of the test into account. Furthermore, Thompson and Green (2013) argue that it might be better to rely on theory and past empirical findings and to be less dependent on empirical methods like the global fit measures and the modification indices when deciding whether to accept or reject a given level of invariance. This issue has not been settled yet.

^5^Whether and to what extent our analysis procedure corresponds with the common concept of using prior knowledge in the same way as in Bayesian statistics is debatable, since our priors actually correspond to an assumption testing of approximate invariance rather than strictly to prior knowledge.

^6^This procedure corresponds to the comparison between the observed variance-covariance matrix (S) and the expected variance-covariance matrix (∑) using maximum likelihood estimation in structural equation modeling (Bollen, 1989).

^7^For a discussion of other fit measures for Bayesian SEM, see Kaplan (2014) and Levy and Choi (2013). Indeed, as Levy and Choi(2013, p. 599) argue, little research has been conducted on the relative merits and limitations of these fit measures to evaluate model comparisons in BSEM.

^8^The raw data is available at the official site of the European Social Survey: http://www.europeansocialsurvey.org/downloadwizard.

^9^Only standard errors and chi-square differ between MLR and FIML.

^10^To identify the model we used the marker variable method (MVM; see Little et al., 2006). We constrained the factor loading of one item to one and its intercept to zero. To test the robustness of our findings, we re-ran the model two more times, and each time with a different item as the marker item. The results remained essentially the same.

^11^When running the Bayesian procedure, we first ran a model where the difference between factor loadings or intercepts across groups has a normal distribution prior with a mean of 0 and a very large variance of 10^10^ (the so-called noninformative prior). This allows us to firstly detect whether there are any calculation problems in the Bayesian analysis (van de Schoot et al., 2013).

^12^After we were unable to achieve partial measurement invariance using the common ways of model fitting, we had to delete countries/time points (groups) based on the modification indices for the exact approach and based on the single group ppp for the approximate approach.

^13^Discussing possible explanations why specific countries are not comparable to others is beyond the scope of the present study. See Davidov et al. (2012) for using multilevel structural equation modeling for explaining noninvariance.

^14^For the single rounds this refers to countries; for all rounds this is combination of countries and time points.

^15^Countries still included are: Belgium 2002–2012; Spain 2002–2006; Finland 2006–2010; United Kingdom 2012; Hungary 2002–2008; Ireland 2008, 2010; Netherlands 2002–2012; Norway 2004–2012; Poland 2006; Portugal 2004–2008; Sweden 2012; Slovenia 2002, 2006.

^16^The partial scalar model corresponds to step 5 in Table 3

^17^An example of the syntax can be found in the Supplementary Material. We would like to thank Bengt and Linda Muthén very much for making it possible to run such a model in the Mplus 7.3 version (Muthén and Muthén, 1998–2014). Previous versions did not allow the inclusion of this number of groups.

^18^A detailed report of the results is beyond the scope of the present study and may be provided by the first author upon request.

^19^Countries/time points not included are Denmark 2002, 2004, 2010, 2012; Spain 2008, 2010, 2012; Finland 2002, 2004; United Kingdom 2010; Hungary 2008; Ireland 2012; Norway 2008; Poland 2008, 2010; Sweden 2012; Slovenia 2010.

^20^To illustrate the comparison, these latent means are based on the model with all countries from the exact test that did not achieve scalar invariance.

^21^In the figure with the Bayesian latent means not all countries and time points are included. Countries/time points which are not included are Denmark 2000, 2002, 2010, 2012; Spain 2008, 2010, 2012; Finland 2000, 2002; United Kingdom 2010; Hungary 2008; Ireland 2012; Norway 2008; Poland 2008, 2010; Sweden 2012; Slovenia 2010.

^22^Note that when certain time points were not available we extrapolated the data.
