# Supplementary material for: The comparability of the universalism value over time and across countries in the European Social Survey: exact vs. approximate measurement invariance
Source: Front Psychol. 2015 Jun 4;6:733. doi: 10.3389/fpsyg.2015.00733 (PMC4455243; doi:10.3389/fpsyg.2015.00733)
Supplement: Supplementary file 1 [file DataSheet1.DOCX]

**Appendix**

Syntax for the approximate model with all countries and time points and an a priori variance of 0.05:

ANALYSIS:

TYPE=MIXTURE;

ESTIMATOR = bayes;

model = allfree;

Bconvergence=0.01;

biterations=500000(20000);

processor=8;

chains is 8;

bseed 100;

MODEL:

%OVERALL%

UN BY ipeqopt@1 ipudrst* impenv* (lam#_1-lam#_3);

[ipeqopt-impenv] (nu#_1-nu#_3);

[ipeqopt@0];

%c#90%

[UN*];

MODEL PRIORS:

do(2,3) diff(nu1_#-nu90_#)~N(0,0.05);

do(2,3) diff(lam1_#-lam90_#)~N(0,0.05);

OUTPUT:

stand Tech1 Tech8;
